# Umbilical hernia as the initial manifestation of pseudomyxoma peritonei: a case report

**DOI:** 10.3389/fsurg.2026.1962278

**Published:** 2026-09-09

**Authors:** Guanjun Shi, Lubiao An, Yiyan Lu, Pu Zhang, Haipeng Zhou, Shi Yan, Ruiqing Ma, Chong Wang

**Affiliations:** 1 Department of Myxoma, Aerospace Center Hospital, Beijing, China; 2 Department of Pathology, Aerospace Center Hospital, Beijing, China

**Keywords:** pseudomyxoma peritonei, umbilical hernia, appendiceal mucinous tumor, cytoreductive surgery (CRS), hyperthermic intraperitoneal chemotherapy (HIPEC), case report

## Abstract

Pseudomyxoma peritonei (PMP) is a rare peritoneal malignancy. Its typical clinical manifestations include abdominal distension, increased abdominal girth, or abdominopelvic masses. It is extremely rare for an umbilical hernia to be the initial manifestation, so this can easily lead to clinical misdiagnosis. A 60-year-old woman presented with a reducible umbilical mass for 10 months. Physical examination revealed marked abdominal distension and a 6-cm firm, irreducible umbilical mass. Serum tumor markers were normal. Computed tomography (CT) showed diffuse low-density abdominopelvic masses consistent with PMP, an appendiceal cystic lesion suggestive of mucinous neoplasm, omental thickening, and periumbilical tumor extension. Ultrasound revealed 9.4-cm ascites with mucin particles and septations. She underwent cytoreductive surgery with hyperthermic intraperitoneal chemotherapy. Intraoperatively, extensive gelatinous mucin, a ruptured cystic appendix, and periumbilical tumor involvement were noted. Postoperative pathology confirmed a high-grade appendiceal mucinous tumor with PMP. The immunohistochemistry results were as follows: cytokeratin 20 positive, caudal type homeobox 2 positive, and Ki-67 labeling index of 30%. Telephone follow-up 3 months after surgery indicated that the patient was doing well. PMP presenting with umbilical hernia as the initial symptom is relatively rare and is often misdiagnosed as a simple umbilical hernia. For middle-aged and elderly female patients with umbilical hernia who have concurrent ascites, thickened peritoneum, or a hard and fixed hernia mass, abdominopelvic CT and ultrasound should be performed to rule out PMP to avoid blindly performing hernia repair and delaying diagnosis and treatment.

## Background

Pseudomyxoma peritonei (PMP) is a rare and complex clinical syndrome characterized by the widespread implantation and metastasis of mucinous tumor cells from various tissue sources throughout the abdominal cavity, continuously producing large amounts of “jelly-like” mucinous ascites (1–3). The literature indicates that the incidence of PMP is approximately 1–3 cases per million people (4–6). Due to the limitations of large-sample epidemiological studies, the actual incidence of PMP may be significantly underestimated. The clinical manifestations of PMP are insidious and lack specificity. It usually manifests as abdominal distension, a progressive increase in abdominal circumference, or abdominal and pelvic masses, while an umbilical hernia as the initial manifestation is exceedingly rare. This article reports a case of PMP with an umbilical hernia as the initial clinical manifestation and retrospectively analyzes its clinical data and treatment process, aiming to provide a reference for the clinical identification of this rare initial manifestation.

## Case presentation

A 60-year-old woman was incidentally found to have a protruding umbilical mass in June 2025. The mass was prominent upon standing or increased intra-abdominal pressure and disappeared in the supine position. The patient was asymptomatic, with no abdominal distension or pain, and initially paid no attention to it. As the umbilical mass gradually enlarged, she presented to a local hospital in January 2026, where an umbilical hernia was suspected and umbilical hernia repair was planned. A preoperative Computed tomography (CT) scan revealed ascites in the abdominal and pelvic cavities, thickening of the peritoneum and greater omentum, and a cystic occupying lesion in the right lower abdomen, suggestive of an appendiceal mucinous neoplasm. Subsequently, a percutaneous biopsy of the umbilical mass was performed, and the pathological result showed mucin lakes within fibroadipose tissue, favoring a diagnosis of pseudomyxoma peritonei.

**Figure 1:**
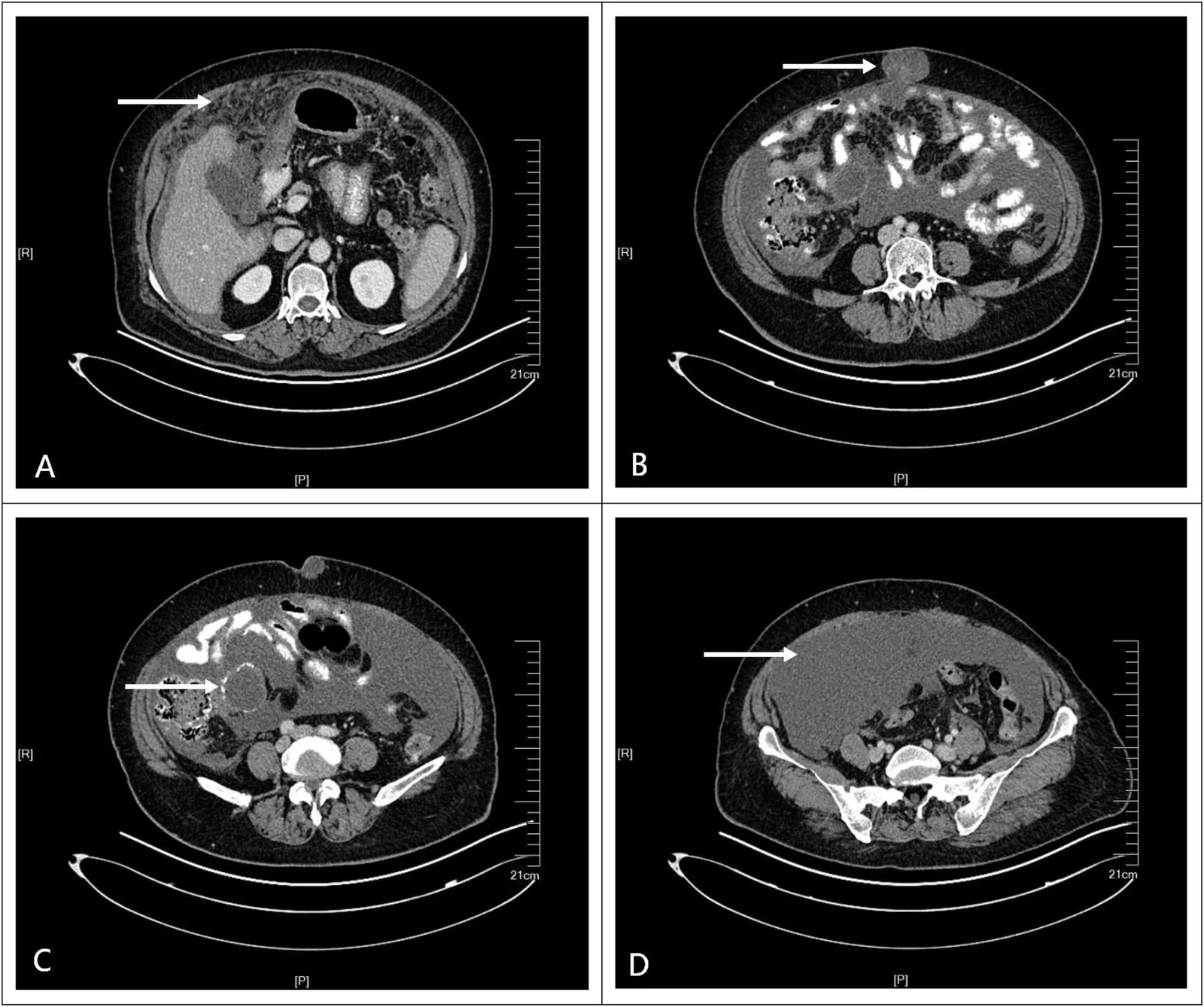
CT: **(A)** Thickened omentum; **(B)** periumbilical mass; **(C)** dilated and enlarged appendix in the right lower quadrant; **(D)** abdominopelvic effusion.

**Figure 2:**
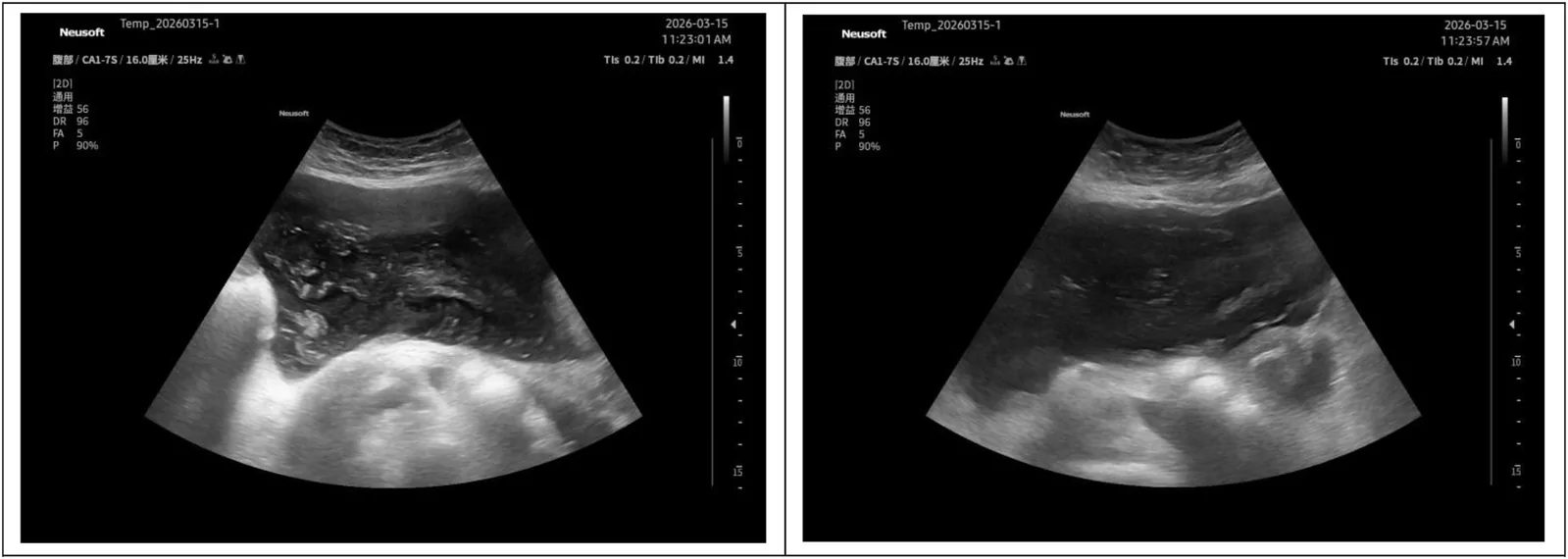
Ultrasound: The abdominopelvic effusion shows poor acoustic transmission, with multiple mucin particles and fibrous septations visible internally.

In April 2026, the patient was admitted to our hospital. Physical examination on admission revealed abdominal distension, with an abdominal circumference of 110 cm. Her abdomen was soft and non-tender, with no rebound tenderness or muscle guarding. A hard, approximately 6-cm mass was palpable at the umbilicus, which did not reduce or disappear upon compression. Shifting dullness was negative. Serum tumor marker levels were as follows: carcinoembryonic antigen (CEA): 1.86 ng/mL; carbohydrate antigen 125 (CA125): 34.23 U/mL; and carbohydrate antigen 19-9 (CA19-9): 26.54 U/mL. Abdominopelvic CT showed diffuse low-density occupying lesions in the abdominal and pelvic cavities, consistent with pseudomyxoma peritonei. A cystic occupying lesion in the appendiceal region was highly suggestive of an appendiceal mucinous neoplasm. The thickening of the greater omentum suggested implantation metastasis. The irregular mass at the umbilicus was considered to be a tumor (Figure 1). Abdominal ultrasonography revealed free fluid in the abdominal and pelvic cavities, with a maximum depth of approximately 9.4 cm, poor acoustic transmission, and multiple small mucin particles with band-like septations (Figure 2).

**Figure 3:**
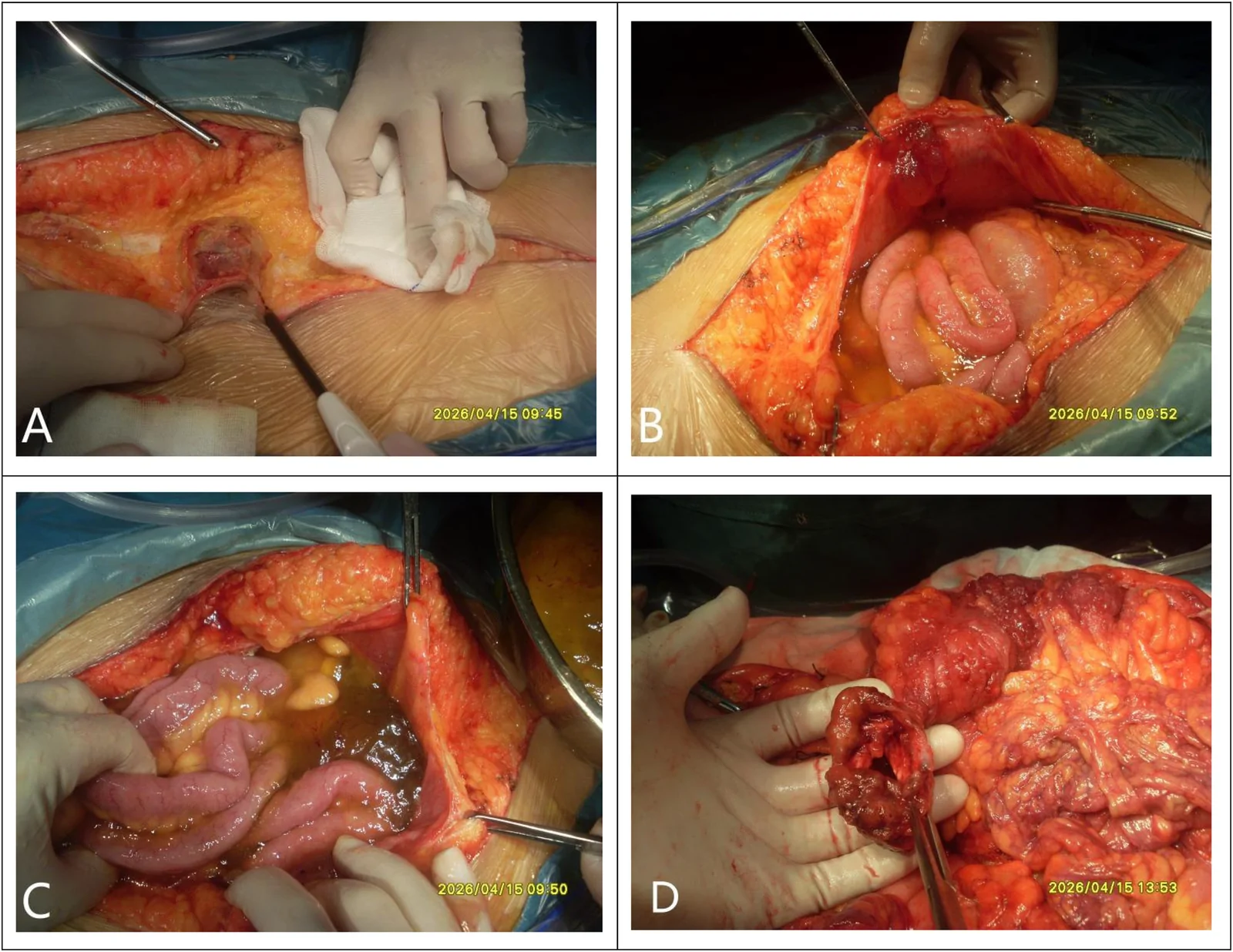
**(A,B)** The tumor is located in the periumbilical tissue and protrudes into the fatty layer. **(C)** Massive gelatinous mucin in the abdominopelvic cavity. **(D)** The appendix is thickened, dilated, and ruptured.

After a comprehensive preoperative evaluation, the patient underwent cytoreductive surgery (CRS) combined with hyperthermic intraperitoneal chemotherapy (HIPEC) under general anesthesia. Intraoperative exploration revealed a large volume of pale-yellow, gelatinous mucus in the abdominal and pelvic cavities, totaling approximately 4,000 mL. The greater omentum was thickened. The umbilicus had been invaded by a mucinous tumor (Figure 3). The appendix was cystically dilated and had ruptured. Diffuse tumor implantation nodules were observed on both parietal peritoneal surfaces, the bilateral diaphragmatic peritoneum, pelvic peritoneum, ovaries, and uterine surface. The peritoneal cancer index was 31. Subsequently, the right hemicolon, appendix, bilateral peritoneal surfaces, pelvic peritoneum, greater omentum, bilateral ovaries, and uterus were resected; the completeness of cytoreduction score was CCR3.

**Figure 4:**
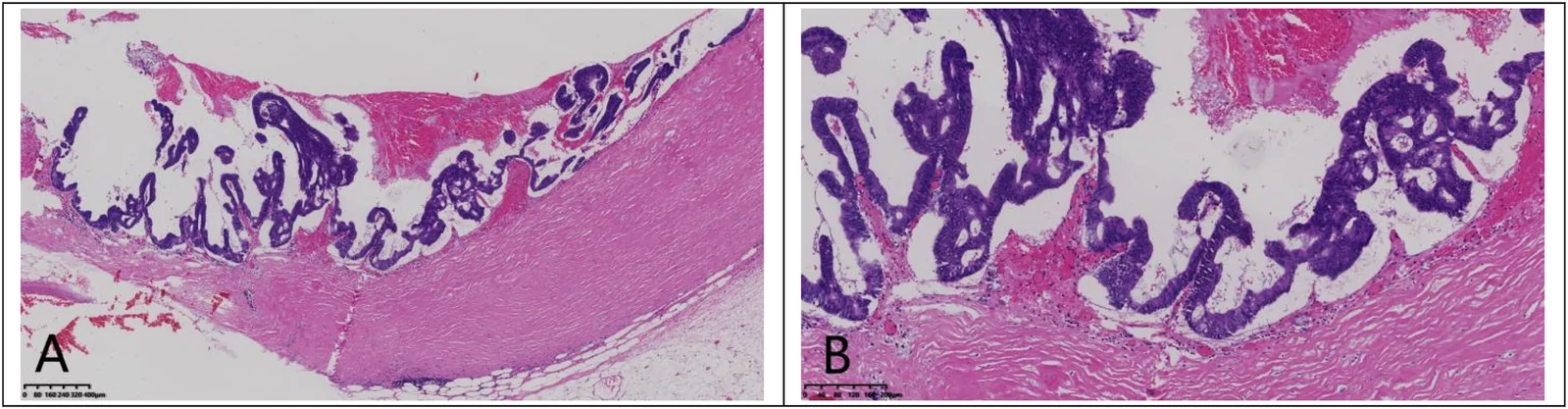
Histopathological findings from the appendix. **(A)** The low-power view shows mucin pools with floating neoplastic glandular structures within the appendiceal wall (HE, ×40). **(B)** The high-power view demonstrates a high-grade mucinous neoplasm with complex cribriform glandular architecture, enlarged nuclei, and marked cytologic atypia (HE, ×100).

**Figure 5:**
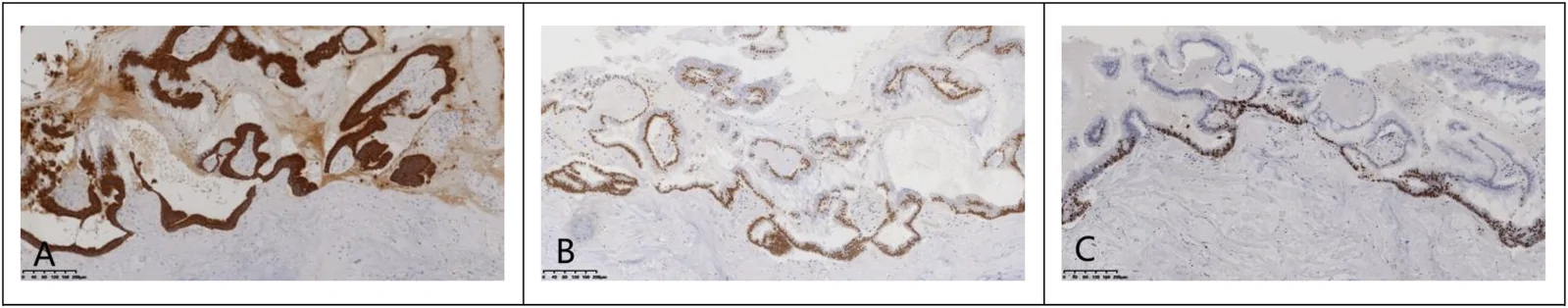
Immunohistochemical findings from the appendix indicate a high-grade appendiceal mucinous neoplasm. Immunohistochemistry shows **(A)** CK20 (+), **(B)** CDX2 (+), and **(C)** a Ki-67 labeling index of 30% (SP staining, ×100).

Postoperative pathology showed a high-grade appendiceal mucinous neoplasm with rupture, accompanied by PMP (Figure 4). Immunohistochemistry revealed cytokeratin 20 positive [CK20(+)], caudal type homeobox 2 positive [CDX2(+)], and Ki-67 labeling index (Ki-67) of 30% (Figure 5).

At the time of writing, the patient has been followed up for 3 months post-treatment, with a favorable recovery status.

## Discussion and conclusions

PMP is a peritoneal malignancy characterized by indolent yet progressively advancing biological behavior. Its clinical manifestations are non-specific, with common symptoms including abdominal distension, increased abdominal girth, abdominal or pelvic masses, and acute appendicitis. Although some patients present with secondary inguinal hernias due to intraperitoneal mucin accumulation and elevated intra-abdominal pressure (7–10), cases with umbilical hernias as the initial manifestation are extremely rare, with few reports in the literature, making them highly susceptible to missed or delayed diagnosis. In the present case, the initial symptom was a typical reducible umbilical mass without abdominal distension, pain, nausea, or other signs of intra-abdominal involvement—features entirely consistent with a simple umbilical hernia, rendering it highly prone to misdiagnosis. As the disease progressed, the patient's umbilical mass evolved from a soft, fully reducible hernial sac in the supine position to a relatively hard, fixed lesion that showed no significant reduction with positional changes. This characteristic transformation in physical findings was not attributable to simple hernia content incarceration but rather to neoplastic tissue infiltration of the periumbilical abdominal wall soft tissues.

Imaging examination is an indispensable tool for the preliminary diagnosis of PMP and provides the core basis for preoperative differential diagnosis. Typical CT findings include diffuse low-density lesions in the abdominal and pelvic cavities. The “scalloped border” sign along the liver and splenic margins carries important diagnostic value for PMP (11). In the present case, preoperative abdominal CT demonstrated mucinous ascites in the abdominal and pelvic cavities, diffuse thickening of the peritoneum and greater omentum, and a cystic occupying lesion in the right lower quadrant appendiceal region—findings highly suggestive of an appendiceal mucinous neoplasm with peritoneal implantation metastasis. Abdominal ultrasonography further revealed intraperitoneal free fluid with a maximum depth of 9.4 cm, poor acoustic transmission, and multiple mucin particles with fibrous septations—representing the characteristic imaging manifestations of mucinous ascites in PMP.

Serum tumor markers have only auxiliary evaluative value for PMP and lack specific diagnostic significance (3). CEA and CA19-9 are primarily used for postoperative disease monitoring and prognostic stratification; however, the majority of patients show only mild elevation, and some cases may fall entirely within the normal range. In this case, CEA, CA125, and CA19-9 all remained within normal limits, further confirming that normal tumor marker levels do not exclude PMP.

In this patient, a percutaneous biopsy of the umbilical mass confirmed the diagnosis. Nevertheless, for patients with imaging findings highly suggestive of PMP, the necessity of a percutaneous biopsy should be carefully evaluated. First, the characteristic gelatinous mucin frequently precludes adequate sample aspiration or identification of typical tumor cells, resulting in a low diagnostic yield. Second, the procedure carries a potential risk of needle tract seeding. Therefore, when characteristic imaging features highly suggestive of PMP are present—including scalloping of the liver margin, septated mucinous ascites, and nodular thickening of the greater omentum—surgical exploration (laparoscopic or open) should be prioritized. This approach enables direct visualization, establishes a definitive histopathological diagnosis, and allows concurrent therapeutic intervention when necessary.

Histopathology combined with immunohistochemistry constitutes the basis for confirming PMP and determining its tissue origin. In this case, percutaneous biopsy of the umbilical mass revealed “mucin lakes within fibroadipose tissue”, which already highly suggested PMP. Postoperative pathology confirmed a high-grade appendiceal mucinous neoplasm with rupture, accompanied by pseudomyxoma peritonei. Immunohistochemistry showed CK20(+), CDX2(+), and Ki-67 of approximately 30%. CDX2 is a highly sensitive and specific marker for intestinal epithelial cells and their neoplasms. Nonaka et al. demonstrated in their study of 42 PMP cases that CDX2 showed diffuse strong positive expression in PMP, and combined detection with CK20 and MUC-2 can effectively confirm the appendiceal origin of PMP (12). The immunophenotype of CK20(+)/CDX2(+) in this case strongly supported a lower gastrointestinal tract (appendiceal) origin, consistent with the postoperative pathological findings. A Ki-67 proliferation index of approximately 30% indicated relatively high proliferative activity of tumor cells. Combined with the pathological diagnosis of a “high-grade” mucinous neoplasm, this suggested relatively aggressive biological behavior in this case.

CRS combined with HIPEC is the current guideline-recommended standard therapeutic strategy for PMP (3, 13, 14). Singh et al. (15) described a young woman who presented with an umbilical hernia accompanied by mucinous discharge as the first clinical manifestation of PMP. Contrast-enhanced CT demonstrated bilateral ovarian mucinous cystadenomas with pseudomyxoma peritonei. She underwent CRS combined with HIPEC and remained free of recurrence at the 1-year postoperative follow-up.

In this case, the patient underwent CRS combined with HIPEC, with intraoperative removal of a large amount of gelatinous mucin and resection of partially involved tissues and organs. However, due to the substantial tumor burden, satisfactory cytoreduction could not be achieved. Although the patient reported favorable recovery during the telephone follow-up at 3 months postoperatively, the long-term outcomes remain to be further evaluated through continued follow-up observation. Given that this case represents a high-grade histological subtype of PMP, postoperative intravenous chemotherapy is recommended, combined with a standardized follow-up protocol—including abdominal CT scans and tumor marker assessments every 6 months—with a minimum follow-up duration of 5 years.

In terms of differential diagnosis, the following conditions should be considered. (1) A simple umbilical hernia: PMP involving the umbilicus may present as a hernia-like protrusion, but the relatively hard consistency and lack of significant positional variation, combined with imaging findings of widespread peritoneal implantation and mucinous ascites, allow for differentiation. (2) Ovarian mucinous tumors: Some cases of PMP may originate from ovarian mature cystic teratomas or ovarian mucinous tumors. However, the immunohistochemical profile of CK20(+)/CDX2(+) in this case strongly supported an appendiceal origin, and both intraoperative and postoperative pathology definitively confirmed the appendix as the primary lesion. (3) Peritoneal malignant mesothelioma: This may also manifest as diffuse peritoneal thickening and ascites, but immunohistochemistry typically expresses mesothelial markers such as Calretinin, WT-1, and D2-40, whereas CK20 and CDX2 are usually negative—findings inconsistent with this case.

This patient with PMP presented with an umbilical hernia as the initial clinical manifestation. During the disease course, the hernial sac progressed from reducible to hard and irreducible, and the patient ultimately underwent CRS combined with HIPEC, with favorable postoperative recovery. However, given the high tumor burden and cytoreduction with macroscopic residual disease, the long-term outcomes remain uncertain and require rigorous, prolonged surveillance.

In conclusion, patients with PMP frequently develop secondary abdominal wall hernias due to significantly elevated intra-abdominal pressure; therefore, clinicians should maintain high vigilance when managing such “hernias”. For middle-aged and elderly female patients with umbilical hernias, if there is concomitant unexplained ascites, thickening of the peritoneum and omentum, or progressively increasing abdominal girth with a hard, poorly reducible hernial mass, direct surgical preparation for an ordinary abdominal wall hernia should be avoided. Instead, abdominal and pelvic CT and ultrasonography should be performed to exclude PMP or other diseases, thereby preventing a blind simple hernia repair that would delay the diagnosis and treatment of the primary mucinous neoplasm.

## Data Availability

The original contributions presented in the study are included in the article/Supplementary Material, further inquiries can be directed to the corresponding authors.
